# The cardiomyopathy of Friedreich's ataxia common in a family: A case report

**DOI:** 10.1016/j.amsu.2021.102408

**Published:** 2021-05-24

**Authors:** Omidreza Amini, Rasool lakziyan, Mahnaz Abavisani, Zohreh Sarchahi

**Affiliations:** a Neyshabur University of Medical Sciences, Neyshabur, Iran; b Rajaie Cardiovascular Medical and Research Center, Iran University of Medical Sciences (IUMS), Tehran, Iran; c Department of Nursing, Gonabad University of Medical Sciences, Gonabad, Iran; d Department of Nursing, Neyshabur University of Medical Sciences, Neyshabur, Iran

**Keywords:** Ataxia Friedrich, Cardiomyopathy, Heart disease

## Abstract

**Introduction and importance:**

Friedreich's Ataxia is an autosomal recessive disease and is usually associated with arterial dysfunction, muscle weakness, spasm in the lower extremities, scoliosis, bladder dysfunction, lack of reflexes in the lower extremities, and imbalance. Approximately 2.3 people have cardiomyopathy. In this article, we have reviewed a case of Friedreich's Ataxia with hypertrophic cardiomyopathy.

**Case presentation:**

A 19-year-old woman with Friedreich's Ataxia has been protesting since she was 11 years old and complained of chest pains, dyspnea, and heart palpitations without a medical history. In ECG, Asymmetrical invert T wave diffuse, diffuse ST-segment depression, and left ventricular hypertrophy were observed. In echocardiography, the left ventricle was reported as hyperimmobile with increased EF (70–75%).

**Clinical discussion:**

In the present study, a patient with Friedrich Ataxia was diagnosed with chest pain, dyspnea, and palpitations without any medical history, and was discharged from the hospital after treatment. In the patients introduced and our patient, there was significant fibro-myocardial hypertrophy, in which the ventricular septal hypertrophy was marked by hypertrophic cardiomyopathy.

**Conclusion:**

Because early diagnosis of the disease is difficult, clinical signs and the patient's current profile at the time of referral will be very helpful.

## Highlights

• Friedreich's Ataxia is an autosomal recessive disease associated with arterial dysfunction.
• early diagnosis of the disease is difficult, clinical signs and the patient's current profile at the time of referral will be very helpful.

## 1 Introduction

Ataxia Friedrich (FRDA) is the most common autosomal recessive spinocerebellar ataxia [1] that occurs due to the development of trinucleotide in the frataxin gene on chromosome 9 [2]. Clinical manifestations include ataxia in the limbs and trunk, arterial dysfunction, lack of deep tendon reflexes, sensory disturbances, skeletal deformity, diabetes, and cardiac involvement [3]. This protein is mitochondrial and is involved in iron homeostasis and respiratory function [4]. Ataxia Friedrich, with a prevalence of 1 in 50,000 people, is the most common inherited degenerative disease of the nervous system and is less common in South Africa, Asia, and the Americas [5]. Neurological symptoms begin before puberty and before the age of 25. The need for a wheelchair is essential for the progression of neuromuscular dysfunction within 10–20 years after the onset of symptoms, and in most cases, neurological symptoms occur before cardiac symptoms. Ataxia-Friedrich's cardiovascular symptoms are less common with concentric hypertrophic cardiomyopathy and with less symmetrical septal hypertrophy. The presence of left ventricular outflow gradient with septal hypertrophy has also been reported. At the same time, dilated cardiomyopathy can occur, which is rare, and dilated cardiomyopathy is caused by the progression of hypertrophic cardiomyopathy. 95% of neurological patients have electrocardiographic and echocardiographic disorders. Wide reverse T wave is common in all leads. About 10% of patients with left ventricular systolic dysfunction have an ejection deficit of less than 50% (1). Severe dilated cardiomyopathy with progressive heart failure can occur [6]. Arrhythmias are less common in Ataxia Friedrich than in high-grade arrhythmias in hypertrophy. Atrial arrhythmias, including fibrillation and fluttering, are associated with progression to dilated cardiomyopathy. Ventricular tachycardia has been reported in dilated cardiomyopathy. Sudden death has been reported but the mechanism is not well established [7]. Idebenone slightly reduces left ventricular mass and hypertrophy, improves left ventricular systolic function, and has no neurological consequences. Cardiac death occurs in patients with dilated cardiomyopathy. Heart failure is the most common cause of death [8]. Complex arrhythmias are responsible for 1.3 mortality due to heart failure in patients. The role of pharmacotherapy or defibrillator therapy in ataxia-Friedrich and dilated cardiomyopathy has not been evaluated, but should be considered as long as treatment for the disease is available [9]. In this article, we have reviewed a case of Friedrich Ataxia with hypertrophic cardiomyopathy.

## 2 Patient introduction

A 19-year-old woman with Friedrich Ataxia, who has been protesting since she was 11, had gone to the emergency room with a complaint of chest pain, dyspnea, and heart palpitations without any previous medical history. He mentioned the background of Friedrich Ataxia in his brother and two of his cousins. The vital signs at the onset of high blood pressure were 120/80, heart rate 120, respiration rate 16, oral body temperature: 36.6 c, and SPO2: 95%.

**Fig. 1:**
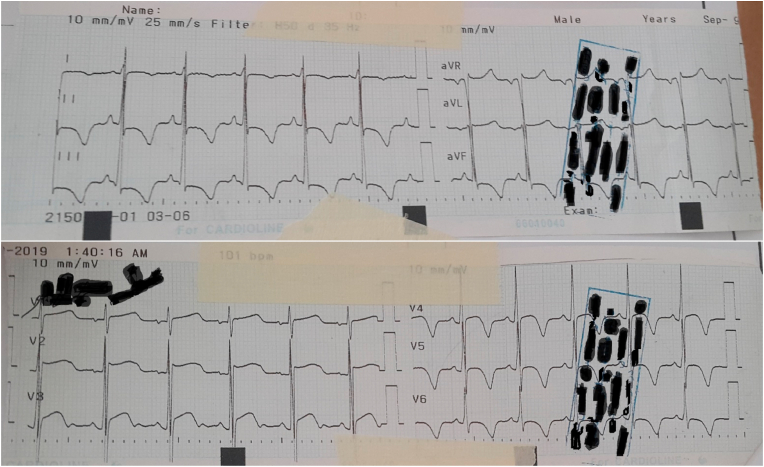
ECGs after entering to hospital emergency ward.

**Fig. 2:**
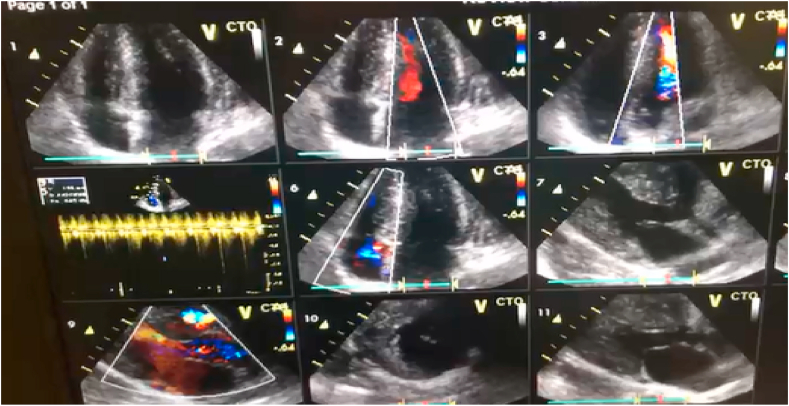
First echocardiography of patient in hospital.

**Fig. 3:**
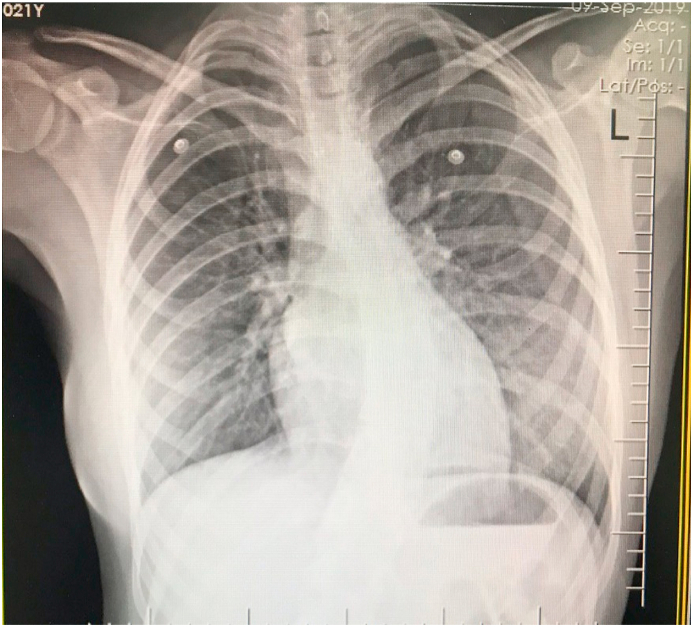
Pulmonary echocardiogram with scoliosis in patient.

The patient was checked by a cardiologist with 10 years’ experience. Troponin tests were negative and other tests were normal. Robotic speech physical examination included arterial dystrophy, gait disturbance, and relative wheelchair dependence. Heart rate S1 and S2 were heard in the cardiac hearing and in the LSB, holosystolic 4.6 was heard. Friction rub was not heard. Lung hearing was normal. In the ECG taken from the patient, incomplete RBBB, J point elevation, Asymmetrical invert T wave diffuse, ST-segment depression diffuse, and according to Cornell and Estes + Romhilt criteria, left ventricular hypertrophy was observed (R III> 20mm + SV3 + R avl> 29mm). In the CXR, the apex is rounded and rises above the diaphragm level, and the sclerosis is quite obvious. Echocardiography showed left ventricular hyperplasia with elevated EF (70–75%), No LVOT obstruction, mild MR, mild TR, severe LVH, increased septal thickness unrelated to HOCM, which was eventually diagnosed with HCM. On complete neurological examination, the reflexes of the Patellar, Achilles, Plantar flexor decreased, and the Romberg test was positive. Magnetic resonance imaging also showed scoliosis of the spine by rotation of the lumbar vertebrae and disc protrusion. In EMG, the performance potential of the sensory nerves was severely reduced, indicating severe polyneuropathy. She was hospitalized for sinus tachycardia and was eventually discharged with beta-blockers (25 mg twice a day) (Fig. 1, Fig. 2, Fig. 3). The present work has been reported in line with the SCARE 2020 criteria [10]:

## 3 Discussion

FRDA is the most common type of inherited ataxia and accounts for approximately 50% of all inherited ataxias [11]. It is a slow, progressive disease in which, according to the FRDA, 15 years after the first symptoms appear, the patient becomes sedentary and the symptoms worsen day by day, and the average age of loss is 3/37 years [12]. Pathological changes in the heart are known in Friedrich Ataxia and were studied by Sanchez et al., In 1976. In most cases, death is due to heart failure and cardiomyopathy. Over the past few years, a large number of hypertrophic cardiomyopathies have been clinically diagnosed in patients with Friedrich Ataxia. Some authors have suggested that hypertrophic cardiomyopathy may be specific to Friedrich Ataxia [13]. Due to the rarity of the disease, the number of articles that reported people with the disease was limited. In one case, an 18-year-old Caucasian girl was diagnosed with Friedrich Ataxia, who first showed signs of her illness at the age of two. Two of the family's eight children as siblings got infected. She was in a wheelchair at the age of 10. At the age of 6, she was diagnosed with left ventricular hypertrophy on her electrocardiogram. Both right and left ventricular diastolic pressures increased to 16 and 22 mm Hg, respectively. In the next years, she complained of palpitations that had never been documented by the E.C.G., and the patient also had no history of shortness of breath or chest pain. One month before his death, he had BP: 65/600 and HR: 80. Lung hearing was okay, and there was no murmur or galloping rhythm in his heart sounds. Chest X-rays showed no evidence of cardiomyopathy or high pulmonary hypertension, but radiographs showed right posterior scoliosis. The electrocardiogram showed scattered changes in the ST and T parts with suspicion of right ventricular hypertrophy and deviation of the right axis. Also, in echocardiography, the systolic and diastolic diameters of the left ventricle were 20 and 30, respectively, and the thickness of the septum and posterior wall was normal. Two days before her death, the patient experienced nausea and vomiting of blood, and progressive and prolonged apnea led to her death. In the following, we will introduce a case of a 22-year-old Caucasian man suffering from Friedreich's Ataxia. Two Venetian sisters and brothers had the same disease, the eldest of whom died at the age of 25. The patient has been dependent on a wheelchair since he was 12 years old. He was recently admitted to the hospital with a fever of over 100° Fahrenheit and a cough. Two days later, the cough and fever continued, and he also experienced a lot of nausea and vomiting. Two days later, the patient became lethargic and pale, had a decreased level of consciousness, and tachycardia. On examination of the cardiovascular condition, HR: 150–180 and irregular, systolic, and jugular veins were non-dilated. The deformed thorax and scoliosis of the back were evident in him. In the ECG taken from the patient, atrial fibrillation with a ventricular velocity of 230 beats per minute was observed with right branch block and severe right axis deviation. The patient died of a continuous shock a few hours after being hospitalized [14]. In the present study, a patient with Friedrich Ataxia was diagnosed with chest pain, dyspnea, and palpitations without any medical history, and was discharged from the hospital after treatment. In the patients introduced and our patient, there was significant fibro-myocardial hypertrophy, in which the ventricular septal hypertrophy was marked by hypertrophic cardiomyopathy.

## 4 Conclusion

Because it is difficult to early diagnose the disease, it can be very helpful to pay attention to the patient's clinical symptoms and current condition at the time of referral.

## Ethical approval

The protocol of the case report of the study was approved by the Research and Ethics Committee of Neyshabur University of Medical Sciences (IR.NUMS.REC.1399.031).

## Sources of funding

None.

## Author contribution

OA: involved in writing an original draft preparation. RL: involved in main clinical part executant. MA: involved in visualization and translation. ZS: involved in supervision. AS: involved in review and editing and involved in project administration.

## Research registration

Not applicable.

## Guarantor

Zohreh Sarchahi.

## Consent

Written informed consent was obtained from the patient for publication of this case report and accompanying images. A copy of the written consent is available for review by the Editor-in-Chief of this journal on request.

## Provenance and peer review

Not commissioned, externally peer-reviewed.

## Declaration of competing interest

None.
